# Intra-arterial injection of Diclofenac by informal health practitioner: A rare complication of a common drug

**DOI:** 10.1016/j.amsu.2022.104736

**Published:** 2022-09-28

**Authors:** Ritvik Chekuri, Manjunath Maruti Pol, Manav Manohar, Bhanu Pradeep Yadav, Raghav Garg

**Affiliations:** Department of Surgical Disciplines, AIIMS, New Delhi, India

**Keywords:** Intra arterial injection, Acute limb isch, a, emia, Limb salvage, Amputation

## Abstract

**Introduction and importance:**

Intra-arterial injections (IA) though rare, cause acute limb ischaemia with often catastrophic outcomes. Symptoms could progress rapidly and early identification and intervention could help in preventing the limb gangrene.

**Methodology:**

The work has been reported in line with the SCARE 2020 criteria:Agha RA, Franchi T, Sohrabi C, Mathew G, for the SCARE Group. The SCARE 2020 Guideline: Updating Consensus Surgical CAse REport (SCARE) Guidelines, International Journal of Surgery 2020; 84:226–230. Operative procedure was performed by consultant of general surgery.

**Case presentation:**

38-year-old male presented to surgery casualty with history of sudden onset of pain and paraesthesia in the left forearm and palm followed by progressive weakness and discolouration, 15 hours following injection of Diclofenac in the mid cubital region.

**Clinical discussion:**

On examination, limb temperature was lower, finger movements were minimal. However, distal pulses were palpable, and duplex ultrasound showed normal triphasic flow. In view of the equivocal clinico-radiological findings, the patient underwent CT–Angiography of upper limb, which showed non-opacification of radial and ulnar arteries. Fasciotomy of forearm, brachial artery exploration and removal of embolus was attempted in a doubtful viable left upper limb. No thrombus was noted. Subsequently, he was managed conservatively, and cervical sympathectomy was done. As there was progressive deterioration in the viability of the limb, the patient underwent an above elbow amputation.

**Conclusion:**

Intra-arterial injections can lead to limb threatening gangrene, the course of which can be rapid A multidisciplinary team approach was necessary to arrive at a diagnosis and provide optimum care.

## Highlights

• An acute pain following a parenteral injection was an ominous sign of intra arterial injection.
• Definitive history and examination should be relied upon rather than becoming dependent on the Duplex Ultrasound findings.
• Delayed presentation was an important risk factor for poor prognosis and limb loss.
• A multidisciplinary team approach was necessary to arrive at a diagnosis and provide optimum care.

## 1 Introduction

Intra-arterial injections (IA) though rare, cause acute limb ischaemia with often catastrophic outcomes. Inadvertent intra arterial injection is rising among drug abusers [1]. Symptoms could progress rapidly from pain, paraesthesia to gangrene and necrosis. Rapid identification and early intervention could help in preventing the limb gangrene. Intra-arterial injections have been described since 1940 [2]. While the initial offending agents were anaesthetic drugs; however, in the last few decades, drug abusers have formed a majority of these cases. Diclofenac as a cause for inadvertent intra arterial injury is very rare.

## 2 Case presentation

38-year-old male visited an informal health practitioner with complaints of burning micturition and abdominal pain. He received injection of Diclofenac in his left cubital fossa. Following this, the patient developed excruciating pain that was sudden in onset and radiating along the borders of the left forearm. He was referred to a nearby hospital, where the Duplex ultrasonography (DUS) was done that did not show any abnormality in the symptomatic arm. The patient was referred to our hospital as the symptoms progressed and were refractory.

The patient arrived at our hospital about 15 hours following injection and presented with complaints of bluish discolouration of left hand, reduced movement in left wrist and fingers, pain in left forearm that increased with digital movements. On examination, the left hand appeared discoloured with delayed capillary filling time. There was reduced temperature in the symptomatic limb when compared to the contralateral limb. The patient maintained flexion of the wrist joint. Brachial, ulnar and radial pulses were palpable. Passive extension was extremely painful to the patient. Saturation of the limb was not recordable in the ipsilateral limb.

## 3 Investigations

**Fig. 1:**
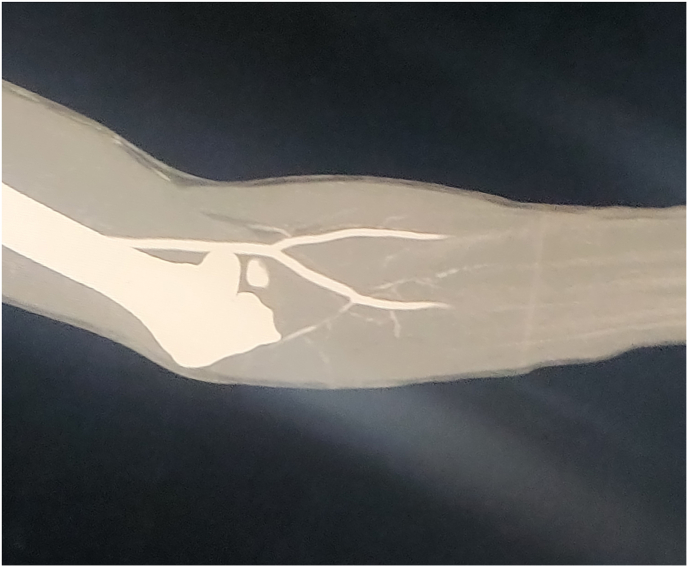
Computed Tomography Angiography image of left upper limb showing non opacification of radial and ulnar artery about 3 cm distal to their origin.

In view of equivocal clinical and DUS findings, a Computed Tomography (CT) Angiography of upper limbs was performed, which showed non-opacification of radial and ulnar arteries beginning about 3 cm distal to their origin. There was no collateral flow (Fig. 1).

## 4 Treatment

Injection Heparin 5000 units was administered intravenous, and the patient had been counselled regarding the prognosis of limb. A provisional diagnosis of left forearm acute limb ischaemia was made, and the patient was taken for surgery. Left forearm fasciotomy was performed, and all muscles examined for their viability. The brachial artery was explored using a 4 French fogarty catheter. However, there was no thrombus noted upon withdrawal of fogarty catheter. Post procedure pulse was not palpable in radial and ulnar artery. Post-operatively, the patient received injection Heparin 1000 units/hour continuous infusion; injection Hydrocortisone 100 mg three times daily; and analgesics and antibiotics. Subsequently, left side cervical sympathectomy was performed on post-operative day-1.

## 5 Outcome and follow-up

**Fig. 2:**
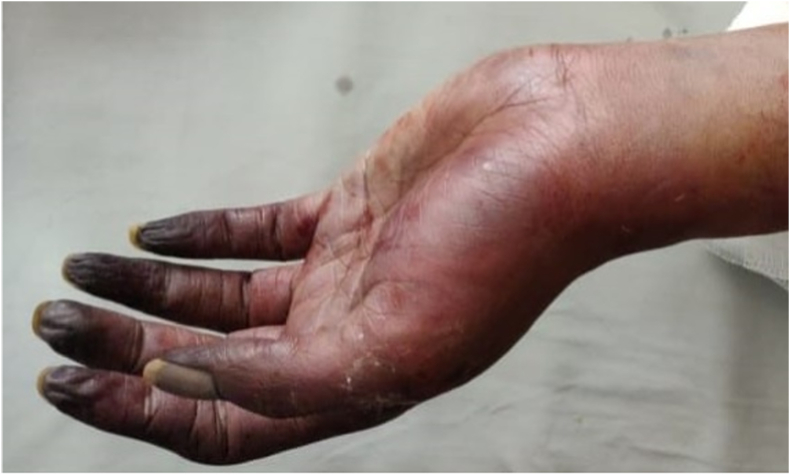
Progression of ischaemia to gangrene seen in left hand on postoperative Day-3.

**Fig. 3:**
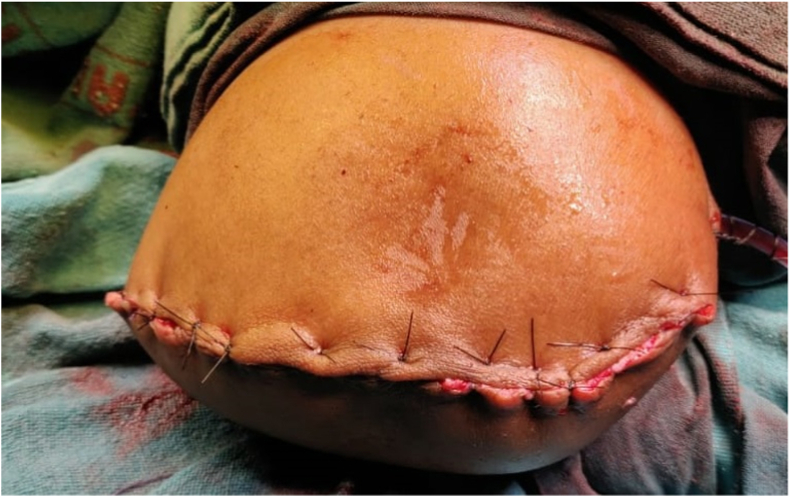
Post-operative picture of above elbow amputation.

On post-operative day-3, the patients’ left limb distal to the cubital fossa was cold with absent radial and ulnar pulsations, absent saturation, and absent digital movements (Fig. 2). The patient underwent left upper limb amputation in view of non-viable left limb. Intraoperatively, there was no bleeding from the forearm muscles, which appeared dead on electro-cautery stimulus. Hence, left side above elbow amputation was done (Fig. 3). The post-operative course following amputation was uneventful, and he was discharged on day-3 following amputation.

## 6 Discussion

Incidences of unintentional IA injections vary from 1 in 56,000 to 1 in 3440 [2,3]. The incidence is difficult to determine accurately because it is an infrequent event. Predisposing factors associated with inadvertent IA injections include, obesity, uncooperative patients during cannulation, history of difficulties in obtaining access, pigmentation of the skin, thoracic outlet syndrome, and pre-existing vascular anomalies can all be considered predisposing factors [4].

Intra-arterial administration of drugs was most commonly reported for Thiopental and Propofol. However, other drugs that have been reported such as Promethazine, Promazine, Floxacillin, Diazepam etc. [[5], [6], [7]].

A few patients have experienced long-term functional hand deficits, temperature hypersensitivity, and paraesthesia even though they didn't have tissue loss. Furthermore, years of therapy and rehabilitation may be needed to overcome some of these sequelae. IA drug injection may cause vessel injury by several mechanisms; for example, the vessel may be obstructed by inert particles, drug crystals, hemolysis, platelet aggregation, vasospasm, venous thrombosis or direct cytotoxicity are the other contributory factors [8].

Many patients complain of immediate discomfort, ranging from local irritation to intense pain distal to the site of injection. Thereafter, many patients have reported sensory (tingling, burning, and paraesthesia) and motor (involuntary muscle contractures and muscle weakness) problems. A few had reported cutaneous manifestations (flushing, mottling). Pain with movement could be an early symptom of compartment syndrome. Eventually, gangrene and permanent functional deficits develop. Apart from functional deficits and disfigurement, many develop chronic pain and complex regional pain syndrome in the affected limb. Sen et al. (2005) reported IA injections related complications that lead to permanent disabilities. These include limb ischaemia, skin necrosis, severe gangrene, and subsequent amputation.

There are no standard guidelines on managing accidental intra-arterial injections. Sen et al. recommended early initiation of anticoagulants in therapeutic doses in all unless contraindicated. Gaspar et al. and Treiman et al. have describing their experiences treating IA drug injection with dexamethasone, heparin, and LMW dextran. However, they reported amputation rate of 26% and 24% respectively [9,10].

A systematic review by Devulapalli et al. (2015) showed that steroids but not anticoagulation showed benefit in lowering amputation rates. Time of presentation was extremely important in saving limb, with 14 hours considered as the upper limit for initiation of intervention in acute limb ischaemia.

Our patient presented to our hospital at the end of 15 hours following symptoms, and underwent fasciotomy apart from this he received steroids, anticoagulants, and cervical sympathectomy. However, delayed presentation might have been the cause for ineffective response and limb loss.

## 7 Learning points/take home messages

• An acute pain following a parenteral injection was an ominous sign of intra-arterial injection.
• Definitive history and examination should be relied upon rather than becoming dependent on the Duplex Ultrasound findings.
• As surgical and non-surgical intervention did not reverse the ill effects of injury caused by the drug, delayed presentation was an important risk factor for poor prognosis and limb loss.
• A multidisciplinary team approach was necessary to arrive at a diagnosis and provide optimum care.

## Ethical approval

Ethical approval has been obtained from Institute Ethics Committee, AIIMS, New Delhi.

## Sources of funding

None.

## Author contributions

Ritvik Chekuri – written the manuscript.

Manjunath Maruti pol – operating surgeon and overall corresponding author.

Manav Manohar-editing the manuscript, collection of data.

Bhanu Pradeep Yadav – photographs, collection of data.

Raghav Garg – collection of data.

## Registration of research studies

This study does not introduce any new surgical techniques or practices. Clinical case reports are not registered in trial registry of India.

## Guarantor

Manav Manohar, MS, AIIMS, New delhi manav2958@gmail.com.

## Consent

Written informed consent was obtained from the patient for publication of this case report and accompanying images.A copy of the written consent is available for review by the Editor-in-Chief of this journal on request.

## Provenance and peer review

Not commissioned, externally peer reviewed.

## Declaration of competing interest

None.

## References

[1] Devulapalli C., Han K.D., Bello R.J. (2015 Nov). Inadvertent intra-arterial drug injections in the upper extremity: systematic review. J. Hand. Surg. Am..

[2] Cohen Sm (1948 Sep 11). Accidental intra-arterial injection of drugs. Lancet.

[3] Sen S., Chini E.N., Brown M.J. (2005 Jun). Complications after unintentional intra-arterial injection of drugs: risks, outcomes, and management strategies. Mayo Clin. Proc..

[4] Ghouri A.F., Mading W., Prabaker K. (2002 Aug). Accidental intraarterial drug injections via intravascular catheters placed on the dorsum of the hand. Anesth. Analg..

[5] Salama T., Aghoutane E.M., Fezzazi R.E. (2016 Dec 6). Gangrène de la main après injection accidentelle intra artérielle de floxacilline: à propos d’un cas [Gangrene of the hand due to accidental intra-arterial injection of floxacilline: about a case]. Pan. Afr. Med. J..

[6] Joist A., Tibesku C.O., Neuber M. (1999 Jun 18). Fingergangrän nach akzidenteller intraarterieller Injektion von Diazepam [Gangrene of the fingers caused by accidental intra-arterial injection of diazepam]. Dtsch. Med. Wochenschr..

[7] Kinmonth Jb, Shepherd Rc (1959 Nov 7). Accidental injection of thiopentone into arteries: studies of pathology and treatment. Br. Med. J..

[8] Rutherford R.B., Baker J.D., Ernst C. (1997 Sep). Recommended standards for reports dealing with lower extremity ischemia: revised version. J. Vasc. Surg..

[9] Gaspar M.R., Hare R.R. (1972 Oct). Gangrene due to intra-arterial injection of drugs by drug addicts. Surgery.

[10] Treiman G.S., Yellin A.E., Weaver F.A. (1990 Oct). An effective treatment protocol for intraarterial drug injection. J. Vasc. Surg..

